# Synthesis and biological evaluation of 1, 3-dihydroxyxanthone mannich base derivatives as anticholinesterase agents

**DOI:** 10.1186/1752-153X-7-78

**Published:** 2013-04-27

**Authors:** Jiangke Qin, Wenli Lan, Zhong Liu, Jun Huang, Huang Tang, Hengshan Wang

**Affiliations:** 1 Key Laboratory of Medicinal Chemical Resources and Molecular Engineering of State Education Ministry, College of chemistry & Chemical Engineering, Guangxi Normal University, Guilin, 541004, P.R. China; 2 Guangzhoujinan Biomedicine Research and Development Center, Guangdong Provincial Key Laboratory of Bioengineering Medicine, Institute of Biomedicine, Jinan University, Guangzhou, 510632, P.R. China

**Keywords:** Xanthone, Mannich bases, Synthesis, Cholinesterase inhibitors

## Abstract

**Background:**

Alzheimer’s disease (AD), a progressive and degenerative disorder, has become one of the severe problems among the aged population all over the world. To use cholinesterase inhibitor drugs has become the most predominant treatment strategy for AD.

**Results:**

A series of novel 1, 3-dihydroxyxanthone Mannich bases derivatives (**1a** ~ **4e**) were synthesized, structure elucidated and evaluated for anti-cholinesterase activity. The result showed that most of the target compounds exhibited moderate to good inhibitory activities with the IC_50_ values at micromole level concentration against both acetylcholinesterase (AChE) and butyrylcholinesterase (BuChE). The preliminary structure-activity indicated that: (i) The alkoxy or alkenoxy substituents in the position 3 of xanthone have a positive influence on the inhibition potency; (ii) types of dialkylamine methyl in position 2 of xanthone affected cholinesterase activities and AChE/BuChE selectivity. Among them, 2-((diethylamino)methyl)-1-hydroxy-3-(3-methylbut-2-enyloxy)-9H-xanthen-9-one showed potent inhibitory activity against AChE with the IC_50_ value of 2.61 ± 0.13 μM and the best inhibitory activity against BuChE with the IC_50_ value of 0.51 ± 0.01 μM. The results of a mixed-type manner in enzyme kinetic experiment and molecular docking study for 2-((diethylamino)methyl)-1-hydroxy-3-(3-methylbut-2-enyloxy)-9H-xanthen-9-one demonstrated that the Mannich base derivatives were likely to bind to the active site (AS) and the peripheral anionic site (PAS) of cholinesterases.

**Conclusions:**

This study suggested that 1, 3-dihydroxyxanthone Mannich base derivatives were potential dual inhibitors of both AChE and BuChE, which may be considered as a kind of novel drug candidates for treatment of AD.

## Background

It is well known that there are two major forms of cholinesterases in the brain of mammals. One is acetylcholinesterase (AChE), it is a special enzyme to hydrolyze the neurotransmitter acetylcholine (ACh). The other is butyrylcholinesterase (BuChE), it is a pseudocholinesterase that remains unanswered, which is less substrate specific for ACh than AChE. However, its expressive level was more abundant in the peripheral system. Both of the two cholinesterase are found in neurons and glial cells as well as in neuritic plaques and tangles in Alzheimer disease (AD) patients [1].

AD is the most common dementia occurs among the elderly people. It is a progressive and neurodegenerative disorder affects regions of the brain that control cognition, memory, language, speech and awareness [2,3]. The two major pathological hallmarks of AD are the progressive loss of cholinergic neural transmission, formation of a beta-amyloid plaques (Aβ-plaques) that forms senile plaques (SPs) and neurofibrillary tangles (NFTs) of hyperphosphorylated tau protein [4,5]. Hence, two hypotheses including cholinergic and β-amyloid [6-8] were developed to interpret this phenomena, which, in essence, states that the cognitive decline in Alzheimer’s disease is due (at least in part) to a loss of cholinergic neurotransmission [9] and the deposition beta-amyloid protein which is toxic to the neuron system. Anti-Aβ therapies are thought to be a pivotal strategy for the cure of AD [10,11].

New findings show that both AChE and BuChE are involved in the breakdown of acetylcholine in the brain. Dual inhibition of these enzymes may increase the efficacy of treatment and broaden the indications. It is demonstrated that AChE has a key role in the acceleration of Aβ-peptide deposition and promoting the formation of Aβ-plaques in Alzheimer’s brain [12]. Recent studies suggest that BuChE is present in key brain areas and may also influence the aggregation of neuritic β-amyloid (Aβ) plaques to form the neurofibrillary plaques causing [13-15].

For clinical purposes, it is particularly important to consider the fact that while brain AChE activity continuously declines, BuChE activity increases continuously during disease progression [16,17]. Clinical data is therefore mounting to suggest that the use of agents with the ability to effectively inhibit BuChE as well as AChE may represent an additional therapeutic strategy for the on-going management of AD [18,19].

To date, there are many drugs had been approved and licensed for curing this disease, such as rivastimine, donepezil and galanthamine. At a certain extent, they are generally considered as cholinesterase inhibitors. Although these drugs share the same therapeutic class, they differ in their pharmacology and pharmacokinetics and possess different degree side effects. Moreover, they just cure mild or moderate degree of AD at the early stage, still no cure for severe type of AD.

Xanthone, chemical name is dibenzo-γ-pyrone, is a basic building block of active component of many naturally medicinal plants. Its derivatives are broadly distributed in the nature, and with a wide range of biological activities, such as anti-bacterial, anti-inflammatory, anti-tumour and *α-*glucosidase inhibitory activities [20-23]. Xanthone derivatives as anti-cholinesterase agents have been received significant attention in recent years. It was reported that xanthone derivatives could inhibit AChE and block the Acetylcholinesterase-induced β-Amyloid aggregation [24-26]. However, there are few reports about the research on xanthone derivates as dual inhibitors of both AChE and BuChE. Recent research showed that macluraxanthone exhibited several hydrophobic interactions and hydrogen bonds with the amino acid residues of the PAS, AS and acyl-binding pocket of AChE and BuChE [27]. Futhermore, Mannich bases have been associated with increased biological potency [28]. So xanthone was used as a building block, and a series of Mannich bases of 1, 3-dihydroxyxanthone analogues with alkoxy and alkenoxy substituted at position 3 of xanthone and dialkylamine methyl substituted at position 2 were designed and synthesized as cholinesterase inhibitors, which possessed dual inhibitory activity to AChE as well as BuChE. Their inhibitory effects on both AChE and BuChE were evaluated. Furthermore, the enzyme kinetic analysis and molecular docking studies were performed to delineate their modes of inhibition.

## Results and discussion

### Chemistry

**Scheme 1:**
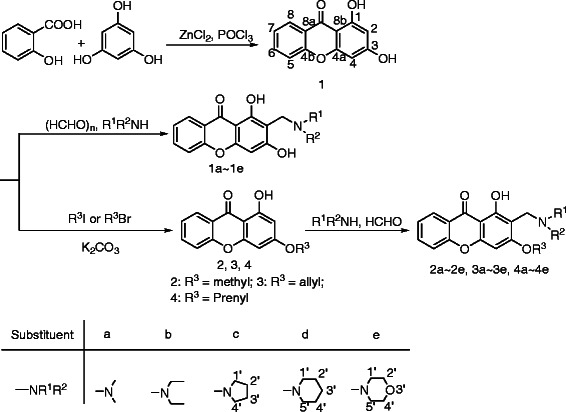
Synthetic route of 1, 3-dihydroxyxanthone mannich base derivatives.

The target compounds **1a ~ 1e**, **2a ~ 2e**, **3a ~ 3e**, **4a ~ 4e** were synthesized according to the synthetic route showed in Scheme 1. 1, 3-dihydroxyxanthone was firstly obtained through the one pot reaction using salicylic acid and phloroglucinol as raw material [29,30]. Then, etherification of the hydroxyl in the position 3 of 1, 3-dihydroxyxanthone was carried out under alkaline condition, three kinds of intermediate compounds **2 ~ 4** with different substituents at position 3 were obtained [31]. Finally, compounds **1 ~ 4** were reacted with formaldehyde and various secondary amines in methanol or acid solution by the Mannich reaction, respectively [32,33], yielded the corresponding Mannich bases of 1, 3-dihydroxyxanthone derivatives.

**Figure 1:**
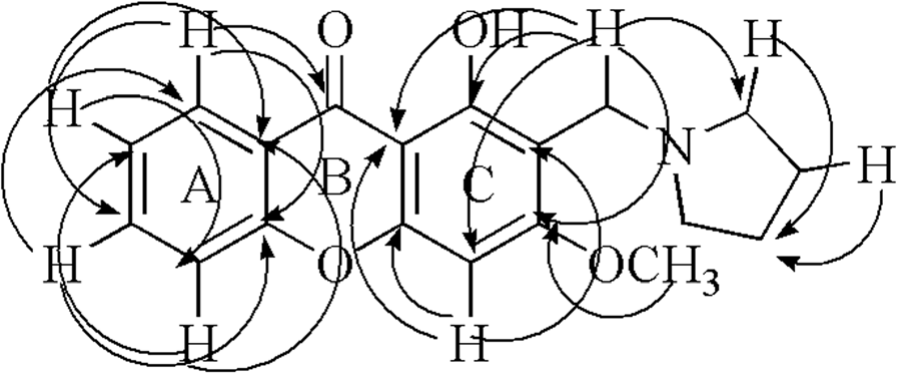
Main connectivities found in the HMBC of Compound 2c.

For the 1, 3-dihydroxyxanthone and the oxygenated derivatives (**2 ~ 4**), 2- and 4-position are active sites and the Mannich reaction may be processed in these two position. In order to establish the exact position of substituents of the title compounds, we hypothesis that if the reaction was carried out in the position 2, the H of CH_2_ inducted by Mannich Reaction correlated with C-1 of xanthone ring in HMBC, if not the H of CH_2_ correlated with C-4a. For validating the hypothesis, **2c** was subjected to HSQC and HMBC spectral analysis. The data was shown in Table 1. It was not difficult to confirm the ^1^H NMR spectral assignments of **2c** by analyzing its coupling relation and comparing its ^1^H NMR data with that of 1, 3-dihydroxyxanthone [34]. In ^1^H NMR, the consecutive aromatic protons of A ring were observed at 8.24 (d, *J* = 7.9 Hz, 1H), 7.35 (t, *J* = 7.5 Hz, 1H), 7.68 (t, *J* = 7.9 Hz, 1H), 7.40 (d, *J* = 8.4 Hz, 1H); the only reserved proton of C ring was observed at 6.43 (s, 1H) which indicated that one of the aromatic protons of C ring (2- or 4- position) was replaced by the Mannich reaction and the inducted CH_2_N groups were observed as singlet at 3.76 ppm. From HSQC, HMBC and also comparing its ^13^C NMR data with that of 1, 3-dihydroxyxanthone [35], its ^13^C NMR assignments could easy be appointed, in which C-1 and C-4a in xanthone core were observed at 161.70, 157.54 respectively. From the data of HMBC, we can observe that the H of CH_2_N correlated with C-1 of xanthone ring, not with C-4a, which confirmed that the Mannich reaction was carried out in the position 2. The main connective found in the HMBC was depicted in Figure 1. Other target compounds’ spectral data was similar to that of **2c** and all the new compounds (**1a** ~ **4e**) were characterized by MS, NMR and IR.

**Table 1:** The NMR data of compound 2c

**Assignment**	^**13**^**C NMR**	^**1**^**H NMR**	**HSQC**	**HMBC**
1	161.70			
2	104.15			
3	165.67			
4	90.09	6.43 (s, 1H)	90.09	157.54, 104.15, 109.21
4a	157.54			
4b	156.21			
5	117.76	7.40 (d, *J* = 8.4 Hz, 1H)	117.74	121.19, 124.27
6	135.07	7.68 (t, *J* = 7.9 Hz, 1H)	135.07	126.27, 156.21
7	124.27	7.35 (t, *J* = 7.5 Hz, 1H)	124.27	117.76, 121.19
8	126.27	8.24 (d, *J* = 7.9 Hz, 1H)	126.27	180.96, 135.07, 156.21
8a	121.19			
9	180.96			
8b	109.21			
OCH_3_	56.37	3.92 (s, 3H)	56.37	165.67
CH_2_N	45.96	3.76 (s, 2H)	45.96	165.67, 161.70, 109.21, 54.24
1' and 4'	54.24	2.63 (brs, 4H)	54.24	23.81
2' and 3'	23.81	1.75 (brs, 4H)	23.81	23.81

### Biological activity

The inhibitory activities of all the synthesized Mannich bases of 1,3-dihydroxyxanthone derivatives against AChE and BuChE *in vitro* were tested by slightly modified Ellman method [36], using acetylthiocholine and butyrylthiocholine iodide as substrates and with Galanthamine · HBr as the reference standard. Inhibition potency of the title compounds toward AChE and BuChE was displayed as IC_50_ values in Table 2.

**Table 2:** Inhibition of AChE and BuChE activities of title compounds


Compd.		R^3^	IC_50_ (μM) ^a^ for AChE	IC_50_ (μM) for BuChE	Selectivity for BuChE/AChE
**1a**		H	106.72 ± 5.78	62.93 ± 0.84	0.59
**1b**		H	73.72 ± 1.36	36.98 ± 0.56	0.50
**1c**		H	81.92 ± 2.04	35.49 ± 1.68	0.43
**1d**		H	79.34 ± 5.78	42.98 ± 1.76	0.54
**1e**		H	95.35 ± 1.70	>100	>1.05
**2a**		CH_3_	2.34 ± 0.20	10.89 ± 0.20	4.66
**2b**		CH_3_	2.40 ± 0.49	6.79 ± 0.56	2.83
**2c**		CH_3_	4.20 ± 0.29	7.21 ± 0.75	1.72
**2d**		CH_3_	7.00 ± 0.07	10.62 ± 0.22	1.53
**2e**		CH_3_	7.76 ± 2.36	>100	>12.89
**3a**			5.84 ± 1.19	4.66 ± 0.64	0.80
**3b**			6.00 ± 0.33	1.54 ± 0.14	0.26
**3c**			4.36 ± 0.58	3.05 ± 0.13	0.70
**3d**			3.70 ± 0.16	1.73 ± 0.08	0.47
**3e**			74.73 ± 4.07	>100	>1.34
**4a**			2.63 ± 0.07	1.49 ± 0.03	0.57
**4b**			2.60 ± 0.13	0.51 ± 0.01	0.20
**4c**			5.85 ± 0.32	2.89 ± 0.07	0.49
**4d**			55.34 ± 8.15	5.61 ± 0.08	0.10
**4e**			24.16 ± 0.40	8.83 ± 0.36	0.37
**Galanthamine · HBr**			1.12 ± 0.02	29.99 ± 0.05	26.78

^a^Data are means ± standard deviation of triplicate independent experiments.

From the data listed in the Table 2, we can clearly see that most of the designed compounds exhibited moderate to good inhibitory activities with the IC_50_ values at micromole level concentration against both of the enzymes.

**Figure 2:**
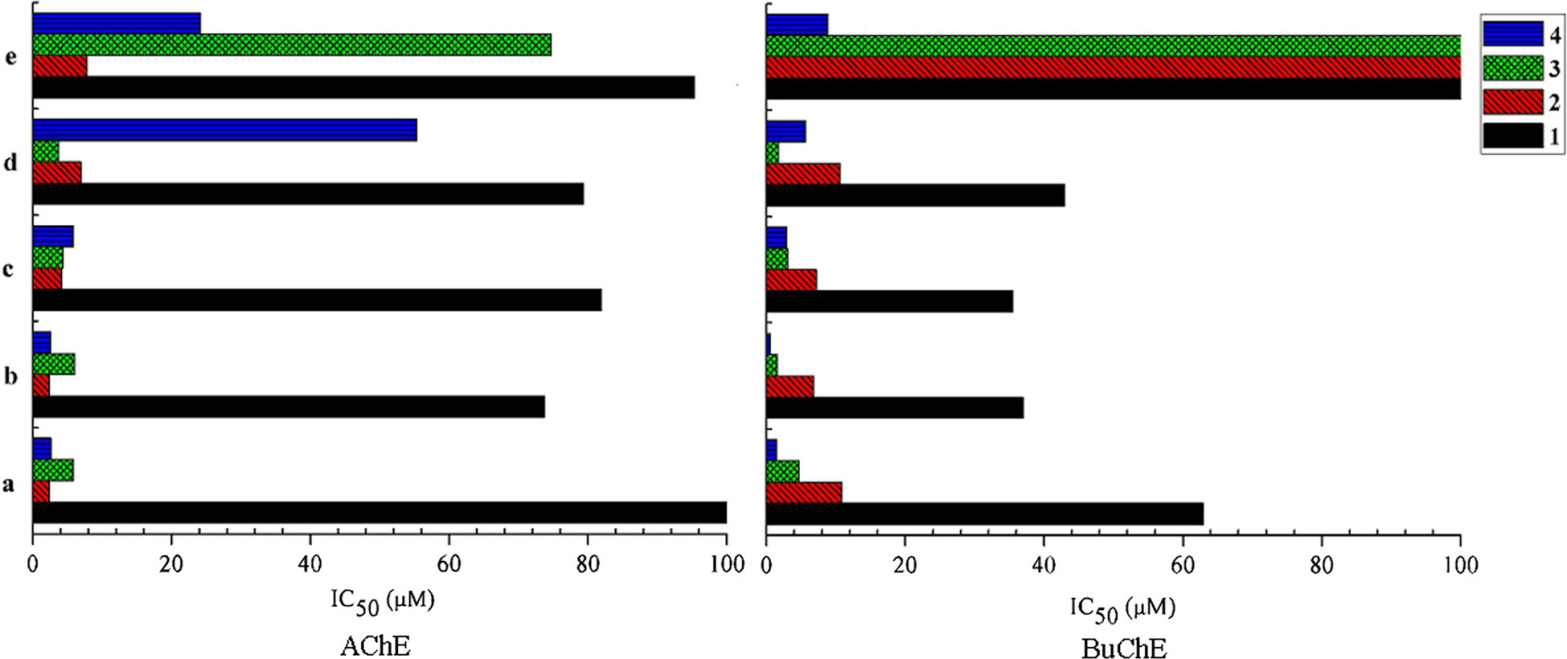
**Effects of substitution in position 2 of xanthone against AChE and BuChE, respectively.** 1, 2, 3, 4 represent hydroxyl, methoxyl, allyloxy, prenylated oxyl substituted in the position 3 of xanthone, respectively. a, b, c, d, e represent diethylamine methyl, dimethylamine methyl, pyrrolidinyl methyl, piperidinyl methyl, morpholino methyl substituted in the position 2 of xanthone, respectively.

Some preliminary structure-activity relationships could be summarized as follows: Whether the variety substituent of amido in the position 2 had influences on anti-cholinesterase activity was firstly assessed. The IC_50_ values were used to draw histogram (Figure 2). As Figure 2 showed, diethylamine methyl in the position 2 of the xanthone exhibited the most potential inhibitory. However, the presence of morpholino methyl showed relatively poor inhibitory activity against both of the enzyme in most case, especially compound **1e**, **2e**, **3e** did not show any inhibition activity at 100 μМ against BuChE. The influence order could be summarized as follows: diethylamine methyl ≈ dimethylamine methyl > pyrrolidinyl methyl > piperidinyl methyl > morpholino methyl against a-cetylcholinesterase activity and diethylamino methyl > pyrrolidinyl methyl > piperidinyl methyl > dimethylamine methyl > morpholino methyl against butyrylcholinesterase activity.

**Figure 3:**
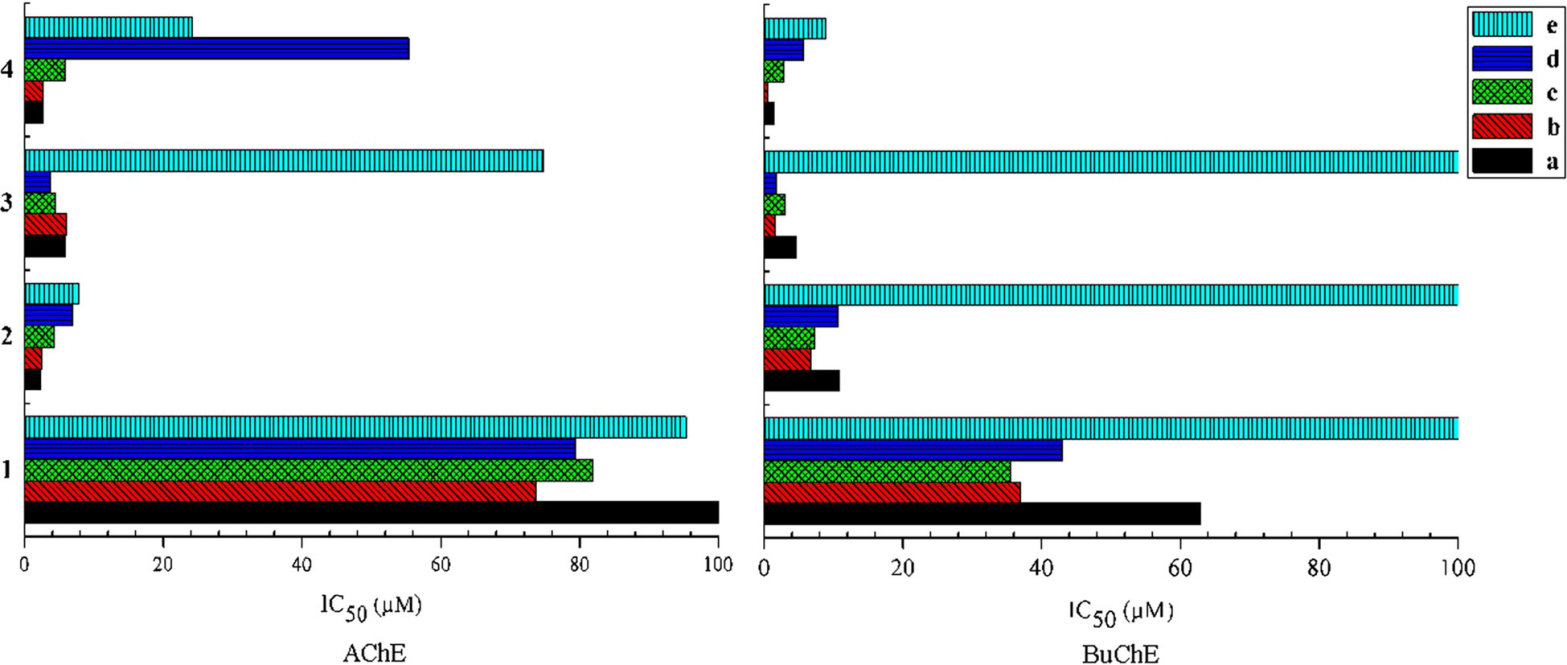
**Effects of substitution in position 3 of xanthone against AChE and BuChE, respectively.** 1, 2, 3, 4 represent hydroxyl, methoxyl, allyloxy, prenylated oxyl substituted in the position 3 of xanthone, respectively. a, b, c, d, e represent diethylamine methyl, dimethylamine methyl, pyrrolidinyl methyl, piperidinyl methyl, morpholino methyl substituted in the position 2 of xanthone, respectively.

The effects of various substituents in the position 3 were subsequently investigated (Figure 3). Firstly, as for the inhibitory activity against AChE, methoxyl substitution shows the most potent inhibitory, which is consistent with a previous report describing that methoxyl was favorable, although the former report focused on the coumarin moiety [37]. The influence order is methoxyl > prenylated oxyl > allyloxy > hydroxyl. Interestingly, as for the anti-butyrylcholinesterase activity, increasing bulkiness of the derivatives, which bear alkenoxy in the position 3 of xanthone exert higher potency. The most active inhibitor against BuChE was **4b** with IC_50_ value of 0.51 ± 0.01 μM, which bears prenyl substituted in the position 3. It also showed potent inhibitory activity against AChE with the IC_50_ value of 2.61 ± 0.13 μM. This result was similar to the Rivera-Becerril’s report [38], confirming the double bond of the prenyl group is interacting as a specific π-hydrogen bond acceptor with the enzyme. The influence order is prenylated oxyl > allyloxy > methoxyl > hydroxyl. In order to explain the different activities between the AChE and BuChE, the ligand binding sites are considered in both enzymes, such as active site and peripheral anionic site (PAS). In terms of the active sites, it is known that the volume of active site in BuChE is larger than AChE [39-41] and hence can accommodate ligands with larger molecular structures. In PAS, there are a number of hydrophobic amino acids, which in AChE are largely aromatic amino acids while in BuChE are largely aliphatic [42-44]. In general, the AChE and BuChE inhibitory activities of etherified compounds with alkyl group in the position 3 are better than those with hydroxyl group. It suggests that hydrophobic circumstance is importance for the anti-cholinesterase activities. The substituents in the position 3 of xanthone show a positive influence on the inhibition potency.

From Table 2, we also could see that most of the title compounds had BuChE/AChE selectivity index in the range of 0.1-0.8, which suggested that these compounds were potential dual inhibitors of both cholinesterases and showed more inhibitory potency toward BuChE. Because bivalent ChE inhibitors or selective BuChE inhibitors represent a new therapeutic strategy for the on-going management of AD [18,19], thus they might become a novel kind of potential drug candidates for AD treatment.

**Figure 4:**
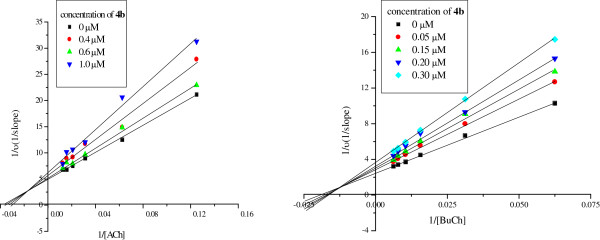
Lineweaver-Burk plots of AChE and BuChE inhibitory activity in the presence of compound 4b.

The inhibition modes of both cholinesterases caused by the selected potent compound **4b** were investigated by the graphical analysis of steady-sate inhibition data (Figure 4). Reciprocal plots (lineweaver-Burk plots) describing compounds **4b** inhibition showed that the point of the curves intersect at the secondary quadrant. This pattern indicated the mixed-type inhibition. The results revealed that these compounds were likely to bind both of the active site and PAS of both cholinesterases.

**Figure 5:**
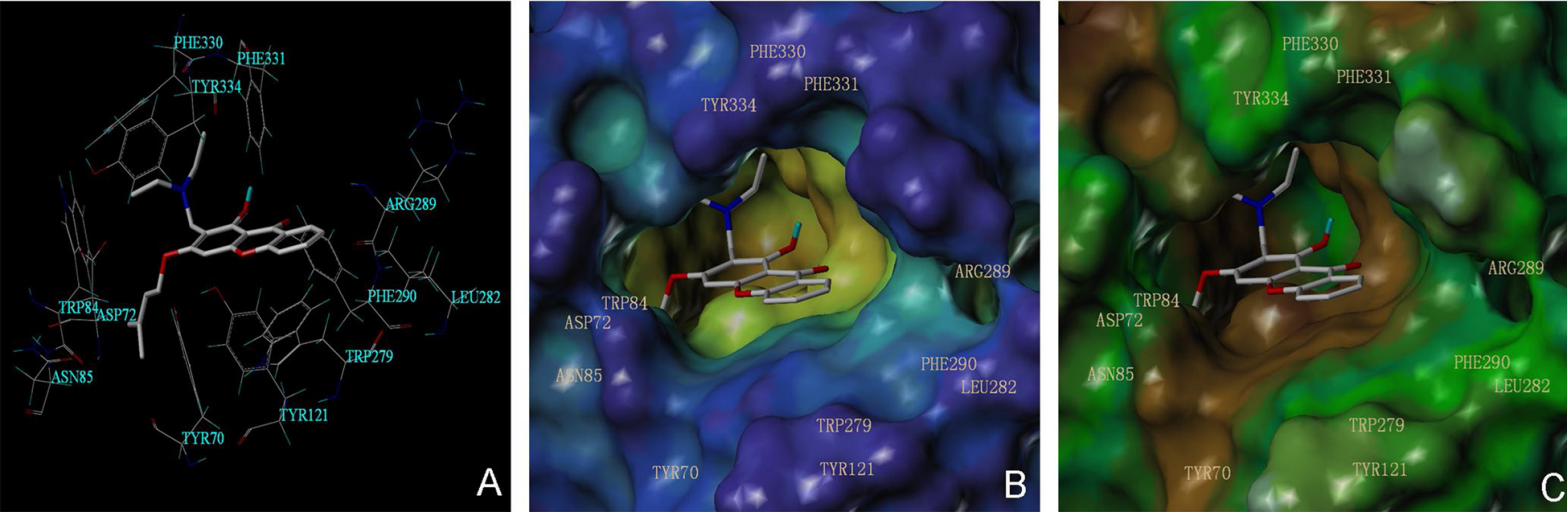
**The binding mode research of compound 4b on AChE by docking simulations.** (**A**) Binding interactions with selected residues of the active site for compound **4b**. (**B**) The MOLCAD surfaces displayed with cavity depth potential of the binding pocket. (**C**) The MOLCAD surfaces of the binding pocket displayed with lipophilic potential.

In order to further investigate the binding modes of Mannich bases derivatives with cholinesterases, we carried out molecular docking studies for the selected potent compound **4b** by Surflex-Dock suite implemented in SYBYL 8.0 software. The PDB codes of 3D crystal structures of human AChE and BuChE are 1EVE and 1P0I, respectively. As shown in Figure 5A, the most energetically favorable binding mode of compound **4b** at the active site of AChE comes into a free binding energy of -7.14 kcal/mol. The MOLCAD surface modeling with cavity depth potential shows that compound **4b** extends into the deep cavity of the binding pocket of AChE (Figure 5B). Compound **4b** occupied to the central hydrophobic region of the binding pocketed arranged by Tyr70, Tyr121, Trp279 and Phe290. It is clearly demonstrated that compound **4b** extends from the anionic subsite of the active site near Trp84 to PAS region near Trp279. Besides, aromatic ring of compound **4b** also forms π-π interactions with Trp279 (Figure 5C). Moreover, the dendrimer-shaped motif at the aromatic ring of compound **4b** may effectively prevent the interaction of catalytic triad of AChE with substrate. These interaction and occupation of compound **4b** with the subsites of AChE explains its mixed inhibition type.

**Figure 6:**
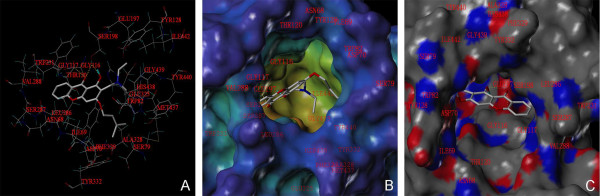
**The binding mode research of compound 4b on BuChE by docking simulations.** (**A**) The binding interactions with selected residues of the active site for compound **4b**. (**B**) The MOLCAD surfaces displayed with cavity depth potential of the binding pocket. (**C**) The MOLCAD hydrogen bonding surfaces of the binding pocket.

On the other hand, Figure 6A illustrates that the binding energy of the most energetically favorable binding mode of compound **4b** towards the active site of BuChE is -8.90 kcal/ mol, consistent with the results of enzyme inhibition assay that compound **4b** is more active to BuChE than to AChE. The MOLCAD cavity depth potential surface also shows that compound **4b** extends into the deep cavity of the binding pocket of BuChE (Figure 6B). Compound **4b** forms three hydrogen bonds with the binding pocket of BuChE. The CO motif of compound **4b** forms a hydrogen bond with the backbone NH of Gly117 and another one with the side chain OH of Ser198, respectively (Figure 6C). In addition, The OH groups at the aromatic ring may also interact with Ser198 by forming a hydrogen bond, leading to the attenuation of catalytic ability of BuChE. These H-bonds are believed to contribute to the higher affinity of compound **4b** towards BuChE, which explains why it is more potent than towards AChE.

## Experimental

### Chemistry

Melting points were measured in X-4 micro-melting point instrument and are uncorrected. IR spectra were taken on Nicolet ESP 360 FI-IR. Direct MS spectra were performed on ESQUIRE HTC instrument in positive mode using KBr pellets. ^1^H NMR and ^13^C NMR spectra were recorded in CDCl_3_ or Acetone on Bruker AVANCE AV 500/125 MHz instruments. Chemical shifts are reported as *δ* ppm using tetramethylsilane (TMS) as the internal standard and couplings expressed in Hertz. Spin multiplicities are given as follows: s (singlet), d (doublet), t (triplet), m (multiplet), or br (broad). Reactions were monitored by thin layer chromatography (TLC) using 0.2 mm Polygram Sil silica gel G254 pre-coated plates with visualization by irradiation with a short-wavelength UV light. Column chromatography was accomplished on Qingdao silica gel (100–200, 200–300 or 300–400 mesh). The process for preparing of title compounds can be found in Additional file 1. HSQC and HMBC spectral analysis of 2c was taken and the data was showed in Table 1.

### Enzyme inhibition assays

Electric-eel AChE (EC 3.1.1.7), horse-serum BuChE (EC3.1.1.8), acetylthiocholine iodide, butyrylthiocholine chloride, 5, 5-dithio-bis-nitrobenzoic acid (DTNB) and Galanthamine hydrobromide (galanthamine · HBr) were purchased from the Sigma. All other agents were analytical grade. AChE and BuChE inhibiting activities were measured by the slight modified spectrophotometric method of Ellman using a 96-well plate reader [36]. Acetylthiocholine iodide and butyrylthiocholine chloride were used as substrates for AChE and BuChE, respectively. The total volume of tested solution in each well was 150 μL. These containing phosphate buffer 118 μL(0.1 M, pH 8.0), DTNB 6 μL(4 mg/mL for AChE or 8 mg/mL for BuChE), different concentration of tested compounds solution 15 μL and AChE or BuChE solution 5 μL, were mixed and incubated for 15 min at 37°C. The reaction was then measured followed by the addition of acetylthiocholine or butyrythiocholine solution (2 mg/mL or 4 mg/mL, respectively) 6 μL. The hydrolysis of acetylthiocholine and butyrylthiocholine were monitored by the formation of yellow 5-thio-2-nitrobenzoate anion as a result of the reaction between DTNB and thiocholine, which released by the hydrolysis of acetylthiocholine and butyrylthiocholine by AChE and BuChE, respectively, at the wavelength of 405 nm for 1 min. Tested compounds and the positive control (Galantha-mine · HBr) were dissolved in DMSO at a concentration of 10 mM before used and diluted in phosphate buffer to the required concentration. All the reactions were performed in triplicate in 96-well microplates in Microplate reader ELX808^™^ (BioTek). The concentrations of tested compounds that inhibited the hydrolysis of substrates (acetylthiocholine and butyrylthiocholine) by 50% (IC_50_) were determined by monitoring the effect of increasing concentrations of these compounds in the assays on the inhibition values. The IC_50_ values were then calculated using the Origin 7.5.

### Enzyme kinetic assays

The enzyme kinetic assays were followed the same method and the similar procedure. The total volume of tested solution in each well was also 150 μL. These containing phosphate buffer 118 μL(0.1 M, pH 8.0), DTNB 6 μL(4 mg/mL for AChE or 8 mg/mL for BuChE), different concentration of tested compounds solution 15 μL and AChE or BuChE solution 5 μL, were mixed and incubated for 15 min at 37°C. The reaction was then measured by the addition of different concentration of (2.0, 1.6, 1.2, 0.8, 0.4, 0.2 mg/mL) acetylthiocholine or (4.0, 3.2, 2.4, 1.6, 0.8, 0.4 mg/mL) butyrythiocholine solution with the volume of 6 μL. The hydrolysis of acetylthiocholine and butyrylthiocholine were monitored in 96-well micro-plates in Microplate reader ELX808^™^ (BioTek) at the wavelength of 405 nm for 1 min. Lineweaver-Burk plots were obtained by plot reciprocal velocity versus substrate.

### Docking studies

Docking studies were performed using the molecular modeling software package SYBYL 8.0 (Tripos, USA). The ligand was charged using the Gasteiger-Huckel and then subjected to energy minimization using the Powell’s method with standard Tripos force field with a 0.01 kcal/(mol*Å) gradient. The minimum-energy structure was used for the subsequent docking calculations. The crystal structures of AChE and BuChE retrieved from the Protein Data Bank (PDB) were used as the initial 3D structure. For AChE, the crystallographic ligand was extracted from the active site, and the resi-dues within a 6.5 Å radius around the enzyme were defined as the active site. For BuChE, the choline, water and cocrystallized small molecules were removed from the p-rotein structure firstly. After the addition of hydrogen and charges, the protein structure was minimized with Amber FF99 force field and 1000 steps. The active site of BuChE was generated by automatic mode implemented in Surflex-Dock program in SYBYL with default parameters. The Surflex-Dock program was used for the docking calculations and MOLCAD surfaces were generated for visualizing the binding mode of the docked protein-ligand complexes.

## Conclusions

In conclusions, a series of Mannich bases of 1, 3-dihydroxyxanthone derivatives were designed, synthesized and subjected to pharmacological evaluation. The results showed that the synthetic compounds possessed good to moderate cholinesterases inhibitory potency. The 1-hydroxy-3-methoxy substituted xanthone derivatives (**2a ~ 2e**) showed higher inhibitory effects on AChE. And the compounds with prenyl substitution in the position 3 of xanthone showed higher BuChE inhibitory potency. In addition, the compounds with diethylamine methyl at the end of the side chain in the position 2 of xanthone possessed higher inhibitory activity. Moreover, the compounds with alkyl substitutions in the position 3 of 1,3-dihydroxyxanthone showed higher potent inhibitory activities compared to **1a** ~ **1e** compounds with hydroxy in the position 3. Kinetic analysis suggested that the the Mannich base compound inhibited both cholinesterases in mixed-type manners, suggesting they might simultaneously interact with the AS and PAS of both enzymes. Molecular docking studies were carried out to further investigate the binding modes of Mannich base derivatives with both cholinesterases, and the result was consistent with that of kinetic analysis. Finally, our results indicated that these new compounds represent useful templates for the development of new anti-AD agents.

## Abbreviations

AD: Alzheimer’s disease; IC50: Concentration producing 50% inhibition; AChE: Acetylcholinesterase; BuChE: Butyrylcholinesterase; AS: Active site; PAS: Peripheral anionic site; Ach: Acetylcholine; Aβ-plaques: Beta-amyloid plaques; SPs: Senile plaques; NFTs: Neurofibrillary tangles; HMBC: Heteronuclear multiple bond connectivity; HSQC: Heteronuclear single quantum correlation; NMR: Nuclear magnetic resonance; MS: Mass spectrometry; IR: Infrared radiation spectroscope; PDB: Protein data bank; 3D: Three dimensional; Tyr: Tyrosine; Trp: Tryptophane; Phe: Phenylalanine; CO: Carbonic Oxide; Gly: Glycine; Ser: Serine; TMS: Tetramethylsilane; CDCl3: Deuterochloroform; TLC: Thinlayer chromatography; UV: Ultraviolet; PE: Petroleum ether; EtOAc: Ethyl acetate; DMSO: Dimethylsulphoxide; APCI-MS: Atmosphere pressure chemical ionization mass spectrometry; DTNB: 5 5-dithio-bis-nitrobenzoic acid

## Competing interests

The authors declare that they have no competing interests.

## Authors’ contributions

The whole project was designed and directed by JQ and HW. WL, JH and HT synthesized and characterized the compounds. The bioassay was perfomed by WL. Molecular docking study was carried out by ZL. JQ wrote the manuscript and HW revised it. All authors read and approved the final manuscript.

## Supplementary Material

Additional file 1 contains the experimental details of preparation and characterization of the relational compounds in the paper. Click here for file
