# Supplementary material for: Synthesis and biological evaluation of 1, 3-dihydroxyxanthone mannich base derivatives as anticholinesterase agents
Source: Chem Cent J. 2013 Apr 27;7:78. doi: 10.1186/1752-153X-7-78 (PMC3673835; doi:10.1186/1752-153X-7-78)
Supplement: Additional file 1 — contains the experimental details of preparation and characterization of the relational compounds in the paper. [file 1752-153X-7-78-S1.doc]

***Procedures for the synthesis of 1, 3-dihydroxyxanthone 1***

To a mixture of salicylic acid (13.8 g , 0.1 mol), phloroglucinol (12.6 g, 0.1 mol), and freshly fused zinc chloride (30 g), phosphoryl chloride (80 mL) was added. The reaction mixture was stirred at 65~70 ˚C for 4~8 h, then slowly poured into crushed ice, and allowed to stand overnight. The solid was collected by filtration, washed with saturated aqueous NaHCO_3_ and water, dried with anhydrous MgSO_4_. The crude products were purified by column chromatography (PE: EtOAc = 4: 1) to afford compound **1** as a yellow solid.

***1,3-dihydroxyxanthone(1)***

11.4 g of 1,3-dihydroxyxanthone was obtained as light yellow solid. Yield: 50 %; m.p. 260~262 ˚C; (reference: 256~258 ˚C [29]); ^1^H NMR (500 MHz, DMSO-*d*_6_), *δ*: 12.80 (s, 1 H, O**H**-1), 11.07 (s, 1 H, 3-O**H**), 8.10 (dd, *J* = 8.0, 1 H, 1.5 Hz, H-8), 7.80~7.83 (m, 1 H, H-6), 7.55 (d, *J* = 8.0 Hz, 1 H, H-5), 7.42~ 7.45 (m, 1 H, H-7), 6.37 (d, *J* =2.1 Hz, 1 H, H-4), 6.20 (d, *J* = 2.1 Hz, 1 H, H-2); IR (KBr), *ν*(cm^–1^): 3327, 1654, 1610, 1570, 1491, 1470, 1445, 1222, 1163, 1078, 827, 762.

***General procedure for the synthesis of 2~4***

To a solution of **1** (4.50 g, 19.7 mmol) in acetone (200 mL), freshly ignited K_2_CO_3_ (3.40 g, 24.6 mmol) was added and followed by methyl iodide, or allyl bromide, or prenyl bromide at room temperature. Each reaction mixture was separately stirred at room temperature or refluxed for 4~8 h. The progress of the reaction was monitored by TLC. On completion, the solvent was removed by filtration, the filtrate was evaporated under reduced pressure and the residue was purified by column chromatography to yield the compounds **2**, **3**, **4**, respectively.

***1-hydroxy-3-methoxy-9H-xanthen-9-one (2)***

Compound **1** and methyl iodide (10 ml, 0.197 mol) were reacted as described above at room temperature. The crude product was purified by column chromatography eluting with PE: EtOAc = 16: 1 to yield compound **2**, and crystallized with acetone as a yellow needle solid. Yield: 84%; m.p. 147~148 ˚C; ^1^H NMR (500 MHz, Acetone-*d*_6_) *δ*: 8.20 (dd, *J*=1.3, 7.8 Hz, 1H, H-8), 7.85 (td, *J*=1.2, 8.5 Hz, 1H, H-6), 7.54 (d, *J*=8.5 Hz, 1H, H-5), 7.47 (t, *J*=7.5 Hz, 1H, H-7), 6.55 (d, *J*=2.0 Hz, 1H, H-4), 6.34 (d, *J*=1.9 Hz, 1H, H-2), 3.95 (s, 3H, OC**H**_3_).

***3-allyloxy-1-hydroxy -9H-xanthen-9-one (3)***

Compound **1** and allyl bromide (2 ml, 23.0 mmol) were reacted as described above at refluxed condition. The crude product was purified by column chromatography eluting with PE: EtOAc = 32: 1 to yield compound **3** as a yellow-white solid. Yield: 74.9%; m.p. 136~137 ˚C; ^1^H NMR (CDCl_3_, 500 MHz) *δ*: 12.85 (brs, 1H, OH), 8.20 (dd, *J*=1.5, 7.9 Hz, 1H, H-8), 7.85 (td, *J*=1.6, 8.7 Hz, 1H, H-6), 7.54 (d, *J*=8.5 Hz, 1H, H-5), 7.47 (t, *J*=7.7 Hz, 1H, H-7), 6.56 (d, *J*=2.1 Hz, 1H, H-4), 6.36 (d, *J*=2.2 Hz, 1H, H-2), 6.07~6.15 (m, 1H, C**H**=), 5.50 (d, *J*=1.5 Hz, 1H, =C**H**_2_), 5.46 (d, *J*=1.6 Hz, 1H, =C**H**_2_), 4.75 (d, *J*=1.5 Hz, 2H, OC**H**_2_);

***1-hydroxy-3-(3-methylbut-2-enyloxy)-9H-xanthen-9-one (4)***

Compound **1** and pre-nyl bromide (2.95 mL, 24 mmol) were reacted as described above at refluxed condition. The crude product was purified by column chromatography eluting with PE: EtO-Ac = 16:1 to yield compound **4** as a yellow-white solid. Yield: 83.6%; m.p. 137~138 ˚C; ^1^H NMR (CDCl_3_, 500 MHz) *δ*: 12.88 (s, 1H, OH), 8.27 (dd, *J*=1.4, 8.0 Hz, 1H, H-8), 7.73 (td, *J*=1.6, 8.6 Hz, 1H, H-6), 7.45 (d, *J*=8.5 Hz, 1H, H-5), 7.40 (t, *J*=7.5 Hz, 1H, H-7), 6.46 (d, *J*=2.1 Hz, 1H, H-4), 6.38 (d, *J*=2.2 Hz, 1H, H-2), 5.53 (t, *J*=6.7 Hz, 1H, C**H**=), 4.63 (d, *J*=6.8 Hz, 2H, OC**H**_2_), 1.85 (s, 3H, =C-C**H**_3_), 1.80 (s, 3H, =C-C**H**_3_);

***General procedure for the preparation of compounds 1a~e***

Paraformaldehyde (60 mg, 2 mmol) was dissolved in methanol (15 mL) at 65 ˚C, treated with corresponding secondary amines (3 mmol), stirred for 1 h. Then a solution of 1, 3- dihydroxyxanthone (0.456 g, 2 mmol) in methanol (10 ml) was added to the reaction mixture. After stirring at reflux for 4~12 h, the progress of the reaction was monitored by TLC. On completion, the reaction mixture was cooled, concentrated under vacuum and then subjected to column chromatography on silica gel yield the compounds ***1a~e*** respectively.

***2-((dimethylamino)methyl)-1,3-dihydroxy-9H-xanthen-9-one (1a)***

Compound **1** and a solution of dimethylamine (0.63 mL, 33%, 3 mmol) were reacted as described above. The crude product was purified by column chromatography eluting with chloroform: methanol = 20: 1 to yield compound **1a** as a yellow solid. Yield: 31.5%; m.p. 148~149 ˚C; ^1^H NMR (CDCl_3_, 500 MHz) *δ*: 8.23 (dd, *J*=2.0, 10.0 Hz, 1H, H-8), 7.68 (td, *J*=2.0, 11.0 Hz, 1H, H-6), 7.40 (d, *J*=10.5 Hz, 1H, H-5), 7.34 (t, *J*=10.0 Hz, 1H, H-7), 6.36 (s, 1H, H-4), 3.89 (s, 2H, C**H**_2_N), 2.45 (s, 6H, 2C**H**_3_); ^13^C NMR (CDCl_3_, 125 MHz) *δ*: 180.53 (C-9), 168.39 (C-3), 160.42 (C-1), 157.39 (C-4a), 155.98 (C-4b), 134.66 (C-6), 125.69 (C-8), 123.72 (C-7), 120.62 (C-8a), 117.54 (C-5), 102.66 (C-8b), 102.34 (C-2), 94.83(C-4), 54.58 (**C**H_3_), 44.19 (**C**H_2_N); IR (KBr) *ν*: 3810, 3450, 2846, 2368, 1651, 1615, 1507, 1467, 1384, 1359, 1322, 1303, 1224, 1173, 1133, 1108, 1078, 1057, 838, 751, 606 cm^–1^; APCI-MS *m/z*: 285.95[M+H]^+^. Anal. calcd for C_16_H_15_NO_4_: C 67.36, H 5.30, N 4.91; found C 67.20, H 5.73, N 4.92.

***2-((diethylamino)methyl)-1,3-dihydroxy-9H-xanthen-9-one (1b)***

Compound **1** and a solution of diethylamine (0.3 mL, 3 mmol) were reacted as described above. The crude product was purified by column chromatography eluting with chloroform: methanol = 40: 1 to yield compound **1b** as a yellow solid. Yield: 24.0%; m.p. 99~100 ˚C; ^1^H NMR (CDCl_3_, 500 MHz) *δ*: 8.22 (d, *J*=8.0 Hz, 1H, H-8), 7.67 (t, *J*=7.2 Hz, 1H, H-6), 7.40 (d, *J*=8.5 Hz, 1H, H-5), 7.34 (t, *J*=7.3 Hz, 1H, H-7), 6.31 (s, 1H, H-4), 3.98 (s, 2H, C**H**_2_N), 2.73~2.81 (m, 4H, 2C**H**_2_), 1.19 (t, *J*=7.3 Hz, 6H, 2C**H**_3_); ^13^C NMR (CDCl_3_, 125 MHz) *δ*: 179.16 (C-9), 168.35 (C-3), 159.13 (C-1), 156.17 (C-4a), 154.81 (C-4b), 133.32 (C-6), 124.48 (C-8), 122.43 (C-7), 119.53 (C-8a), 116.32 (C-5), 101.35 (C-8b), 100.91 (C-2), 93.79 (C-4), 48.29 (2**C**H_2_CH_3_), 45.37 (**C**H_2_N), 9.14 (2**C**H_3_); IR (KBr) *ν*: 3840, 3741, 3439, 2950, 1648, 1613, 1539, 1465, 1358, 1322, 1226, 1173, 1078, 828, 757, 603 cm^–1^; APCI-MS *m/z*: 314.05[M+H]^+^. Anal. calcd for C_18_H_19_NO_4_: C 68.99, H 6.11, N 4.47; found C 68.89, H 6.12, N 4.48.

***1,3-dihydroxy-2-(pyrrolidin-1-ylmethyl)-9H-xanthen-9-one (1c)***

Compound **1** and a solution of pyrrolidine (0.25 mL, 3 mmol) were reacted as described above. The crude product was purified by column chromatography eluting with chloroform: methanol = 40: 1 to yield compound **1c** as a yellow solid. Yield: 19.3%; m.p. 174~176 ˚C; ^1^H NMR (CDCl_3_, 500 MHz) *δ*: 8.24 (d, *J*=7.9 Hz, 1H, H-8), 7.68 (td, *J*=1.61, 9.2 Hz, 1H, H-6), 7.41 (d, *J*=8.4 Hz, 1H, H-5), 7.35 (td, *J*=0.51, 7.5 Hz, 1H, H-7), 6.33 (s, 1H, H-4), 4.06 (s, 2H, C**H**_2_N), 2.83 (brs, 4H, H-1' and 4'), 1.91~1.96 (m, 4H, H-2' and 3'); ^13^C NMR (CDCl_3_, 125 MHz) *δ*: 180.34 (C-9), 169.12 (C-3), 160.01 (C-1), 157.36 (C-4a), 155.97 (C-4b), 134.50 (C-6), 125.65 (C-8), 123.61 (C-7), 120.67 (C-8a), 117.48 (C-5), 103.19 (C-8b), 102.08 (C-2), 94.87 (C-4), 53.48 (C-1' and 4'), 51.05 (**C**H_2_N), 23.73 (C-2' and 3'); IR (KBr) *ν*: 3427, 2102, 1640, 1596, 1539, 1463, 1356, 1333, 1288, 1223, 1169, 1146, 1100, 989, 844, 745, 590 cm^–1^; APCI-MS *m/z*: 312.01[M+H]^+^. Anal. calcd for C_18_H_17_NO_4_: C 69.44, H 5.50, N 4.50; found C 69.34, H 5.51, N 4.49 .

***1,3-dihydroxy-2-(piperidin-1-ylmethyl)-9H-xanthen-9-one (1d)***

Compound **1** and piperidine (0.3 mL, 3 mmol) were reacted as described above. The crude product was purified by column chromatography eluting with chloroform: methanol = 40: 1 to yield compound **1d** as a yellow solid. Yield: 27.7%; m.p. 147~149 ˚C; ^1^H NMR (CDCl_3_, 500 MHz) *δ*: 8.24 (d, *J*=7.9 Hz, 1H, H-8), 7.69 (td, *J*=1.09, 8.34 Hz, 1H, H-6), 7.42 (d, *J*=8.4 Hz, 1H, H-5), 7.36 (t, *J*=7.7 Hz, 1H, H-7), 6.34 (s, 1H, H-4), 3.90 (s, 2H, C**H**_2_N), 2.27 (brs, 4H, H-1' and 5'), 1.72 (brs, 6H, H-2', 3'and 4'); ^13^C NMR (CDCl_3_, 125 MHz) *δ*: 180.41 (C-9), 168.81 (C-3), 160.43 (C-1), 157.28 (C-4a), 155.98 (C-4b), 134.58 (C-6), 125.69 (C-8), 123.66 (C-7), 120.67 (C-8a), 117.53 (C-5), 102.25 (C-8b), 101.99 (C-2), 94.88 (C-4), 54.13 (C-1' and 5'), 53.70 (**C**H_2_N), 25.54 (C-2' and 4'), 23.67 (C-3'); IR (KBr) *ν*: 3436, 2937, 1650, 1614, 1464, 1396, 1352, 1225, 1152, 828, 760, 613 cm^–1^; APCI-MS *m/z*: 326.03[M+H]^+^. Anal. calcd for C_19_H_19_NO_4_: C 70.14, H 5.89, N 4.31; found C 70.10, H 5.90, N 4.32 .

***1,3-dihydroxy-2-(morpholinomethyl)-9H-xanthen-9-one (1e)***

Compound **1** and morpholine (0.26 mL, 3 mmol) were reacted as described above. The crude product was purified by column chromatography eluting with PE: EtOAc = 16: 1 to yield compound **1e** as a yellow solid. Yield: 10.0 %; m.p. 172~175 ˚C; ^1^H NMR (CDCl_3_, 500 MHz) *δ*: 8.24 (d, *J*=7.9 Hz, 1H, H-8), 7.72 (t, *J*=7.2 Hz, 1H, H-6), 7.44 (d, *J*=7.7 Hz, 1H, H-5), 7.39 (t, *J*=7.8 Hz, 1H, H-7), 6.29 (s, 1H, H-4), 4.03 (s, 2H, C**H**_2_N), 3.82 (brs, 4H, H-2' and 4'), 2.72 (brs, 4H, H-1' and 5'); ^13^C NMR (CDCl_3_, 125 MHz) *δ*: 180.56 (C-9), 166.80 (C-3), 163.15 (C-1), 155.61 (C-4a), 154.72 (C-4b), 134.77 (C-6), 125.89 (C-8), 124.12 (C-7), 120.55 (C-8a), 117.37 (C-5), 103.12 (C-8b), 99.24 (C-2), 97.79 (C-4), 66.79 (C-2' and 4'), 54.01 (C-1' and 5'), 52.96 (**C**H_2_N); IR (KBr) *ν*: 3436, 2850, 2363, 1643, 1615, 1573, 1442, 1419, 1345, 1330, 1289, 1229, 1173, 1145, 1117, 1074, 986, 830, 760 cm^–1^; APCI-MS *m/z*: 327.99[M+H]^+^. Anal. calcd for C_18_H_17_NO_5_: C 66.05, H 5.23, N 4.28; found C 66.21, H 5.24, N 4.27.

***General procedure for the preparation of compounds 2a~2e, 3a~3e, 4a~4e***

The selected secondary amine was cooled in the ice-water bath for about 5 min and follo-wed to dropwisely add with formaldehyde solution (1 ml, 0.0133 moles, 37~40%), gl-acial acetic acid (10 ml), then the reaction mixture was stirred for about 1 h at room temperature, treated with **2**, **3**, **4**, respectively. Stirring at room temperature for about 1 ~6 d or at 65 ˚C for about 1~4 h, then treated with water (50 ml), continued stirring for about 4~6 h, filtered. The filtrate was stirred and treated with NaOH (10%) until t-he pH was 9~10, the precipitate was filtered, washed with water, dried and then subjected to column chromatography on silica gel yield the compounds ***2a~4e*** respectively.

***2-((dimethylamino)methyl)-1-hydroxy-3-methoxy-9H-xanthen-9-one(2a)***

Compound **2** (0.484 g, 2 mmol) and a solution of dimethylamine (2 mL, 33 %, 8.7 mmol) were reacted as described above. The crude product was purified by column chromatography eluting with chloroform: methanol: ammonia = 40: 1: 0.5 to yield compound **2a** as a yellow solid. Yield: 87.5%; m.p. 131~132 ˚C; ^1^H NMR (CDCl_3_, 500 MHz) *δ*: 8.29 (d, *J*=7.9 Hz, 1H, H-8), 7.74 (t, *J*=7.6 Hz, 1H, H-6), 7.46 (d, *J*=8.4 Hz, 1H, H-5), 7.41 (t, *J*=7.4 Hz, 1H, H-7), 6.50 (s, 1H, H-4), 3.99 (s, 3H, OC**H**_3_), 3.71 (s, 2H, C**H**_2_N), 2.43 (s, 6H, 2C**H**_3_); ^13^C NMR (CDCl_3_, 125 MHz) *δ*: 180.76 (C-9), 165.55 (C-3), 161.67 (C-1), 157.68 (C-4a), 155.93 (C-4b), 134.95 (C-6), 125.97 (C-8), 124.13 (C-7), 120.81 (C-8a), 117.51 (C-5), 108.31 (C-8b), 103.73 (C-2), 90.00 (C-4), 56.33 (O**C**H_3_), 49.47 (2**C**H_3_), 44.90 (**C**H_2_N); IR (KBr) *ν*: 3423, 2939, 1645, 1605, 1569, 1481, 1467, 1318, 1259, 1230, 1201, 1132, 1097, 762 cm^–1^; APCI-MS *m/z*: 300.08[M+H]^+^. Anal. calcd for C_17_H_17_NO_4_: C 68.21, H 5.72, N 4.68; found C 68.15, H 5.72, N 4.67 .

***2-((diethylamino)methyl)-1-hydroxy-3-methoxy-9H-xanthen-9-one (2b)***

Compound **2** (0.484 g, 2 mmol) and diethylamine (1 mL, 9.70 mmol) were reacted as described a-bove. The crude product was purified by column chromatography eluting with chloroform: methanol: ammonia = 40: 1: 0.5 to yield compound **2b** as a yellow solid. Yield: 59.9%; m.p. 132~135 ˚C; ^1^H NMR (CDCl_3_, 500 MHz) *δ*: 8.27 (dd, *J*=1.4, 7.9 Hz, 1H, H-8), 7.70 (td, *J*=1.3, 7.7 Hz, 1H, H-6), 7.42 (d, *J*=8.4 Hz, 1H, H-5), 7.37 (t, *J*=7.7 Hz, 1H, H-7), 6.45 (s, 1H, H-4), 3.94 (s, 3H, OC**H**_3_), 3.71 (s, 2H, C**H**_2_N), 2.67 (q, *J*=7.0 Hz, 4H, 2C**H**_2_CH_3_), 1.13 (t, *J*=7.1 Hz, 6H, 2CH_2_C**H**_3_); ^13^C NMR (CDCl_3_, 125 MHz) *δ*: 180.98 (C-9), 166.03 (C-3), 162.25 (C-1), 157.97 (C-4a), 156.47 (C-4b), 135.27 (C-6), 126.64 (C-8), 124.53 (C-7), 121.65 (C-8a), 118.01 (C-5), 109.61 (C-8b), 104.61 (C-2), 90.45 (C-4), 56.69 (O**C**H_3_), 47.48 (2**C**H_2_CH_3_), 44.40 (**C**H_2_N), 11.69 (2**C**H_3_); IR (KBr) *ν*: 3411, 2969, 1655, 1607, 1569, 1484, 1468, 1398, 1321, 1262, 1230, 1198, 1142, 1091, 793, 603 cm^–1^; APCI-MS *m/z*: 328.14[M+H]^+^. Anal. calcd for C_19_H_21_NO_4_: C 69.71, H 6.47, N 4.28; found C 69.84, H 6.46, N 4.29.

***1-hydroxy-3-methoxy-2-(pyrrolidin-1-ylmethyl)-9H-xanthen-9-one (2c)***

Compound **2**(0.484 g, 2 mmol) and pyrrolidine(1 mL, 12.1 mmol) were reacted as described above. The crude product was purified by column chromatography eluting with chloroform: methanol: ammonia = 40: 1: 0.5 to yield compound **2c** as a yellow solid. Yield: 89.9%; m.p. 161~163 ˚C; IR (KBr) *ν*: 3425, 2965, 2790, 1652, 1622, 1492, 1468, 1443, 1394, 1318, 1265, 1232, 1206, 1144, 1124, 833, 792, 749, 603 cm^–1^; APCI-MS *m/z*: 326.14[M+H]^+^. Anal. calcd for C_19_H_19_NO_4_: C 70.14, H 5.89, N 4.31; found C 70.10, H 5.88, N 4.31. The NMR (CDCl_3_; ^1^H NMR: 500 MHz; ^13^C NMR: 125 MHz) datas of **2c** were showed in Table 1.

***1-hydroxy-3-methoxy-2-(piperidin-1-ylmethyl)-9H-xanthen-9-one(2d)***

Compound **2** (0.484 g, 2 mmol) and piperidine (1 mL, 10.1 mmol) were reacted as described above. The crude product was purified by column chromatography eluting with chloroform: methanol: ammonia = 40: 1: 0.5 to yield compound **2d** as a yellow solid. Yield: 80.5%; m.p. 166~168 ˚C; ^1^H NMR (CDCl_3_, 500 MHz) *δ*: 8.25 (d, *J*=7.9 Hz, 1H, H-8), 7.68 (t, *J*=7.7 Hz, 1H, H-6), 7.40 (d, *J*=8.4 Hz, 1H, H-5), 7.35 (t, *J*=7.7 Hz, 1H, H-7), 6.43 (s, 1H, H-4), 3.92 (s, 3H, OC**H**_3_), 3.65 (s, 2H, C**H**_2_N), 2.52 (brs, 4H, 1' and 5'), 1.57 (brs, 4H, 2' and 4'), 1.40 (brs, 2H, 3'); ^13^C NMR (CDCl_3_, 125 MHz) *δ*: 181.06 (C-9), 166.11 (C-3), 162.26 (C-1), 157.85 (C-4a), 156.45 (C-4b), 135.28 (C-6), 126.57 (C-8), 124.51 (C-7), 121.56 (C-8a), 117.80 (C-5), 108.78 (C-8b), 104.48 (C-2), 90.33 (C-4), 56.62 (O**C**H_3_), 54.85 (C-1' and 5'), 50.10 (**C**H_2_N), 26.66 (C-2' or 4'), 26.50 (C-2' or 4'), 24.83 (C-3'); IR (KBr) *ν*: 3426, 2938, 2846, 1650, 1608, 1485, 1468, 1448, 1398, 1374, 1318, 1295, 1276, 1249, 1230, 1150, 1133, 1109, 1094, 1036, 1013, 988, 837, 796, 756, 605 cm^–1^; APCI-MS *m/z*: 340.05[M+H]^+^. Anal. calcd for C_20_H_21_NO_4_: C 70.78, H 6.24, N 4.13; found C 70.64, H 6.25, N 4.14.

***1-hydroxy-3-methoxy-2-(morpholinomethyl)-9H-xanthen-9-one(2e)***

Compound **2** (0.484 g, 2 mmol) and morpholine (1 mL, 17.2 mmol) were reacted as described above. The crude product was purified by column chromatography eluting with chloroform: methanol: ammonia = 40: 1: 0.5 to yield compound **2e** as a yellow solid. Yield: 89.0%; m.p. 175~177 ˚C; ^1^H NMR (CDCl_3_, 500 MHz) *δ*: 8.25 (dd, *J*=1.3, 7.9 Hz, 1H, H-8), 7.71 (td, *J*=1.6, 7.7 Hz, 1H, H-6), 7.42 (d, *J*=8.4 Hz, 1H, H-5), 7.38 (t, *J*=7.8 Hz, 1H, H-7), 6.45 (s, 1H, H-4), 3.94 (s, 3H, OC**H**_3_), 3.78 ~ 3.68 (m, 6H, C**H**_2_N, 2' and 4'), 2.58 (t, *J*=7.8, 4H, 1' and 5'); ^13^C NMR (CDCl_3_, 125 MHz) *δ*: 181.35 (C-9), 166.17 (C-3), 162.25 (C-1), 157.94 (C-4a), 156.50 (C-4b), 135.47 (C-6), 126.55 (C-8), 124.65 (C-7), 121.41 (C-8a), 118.21 (C-5), 108.21 (C-8b), 104.36 (C-2), 90.42 (C-4), 67.65 (C-2' and 4'), 56.67 (O**C**H_3_), 54.00 (C-1' and 5'), 49.55(**C**H_2_N); IR (KBr) *ν*: 3421.5, 2945.0, 2853.8, 2340.6, 1655.0, 1607.9, 1570.7, 1483.4, 1466.6, 1397.7, 1316.8, 1288.6, 1231.6, 1207.5, 1136.4, 1114.0, 1094.9, 1036.8, 861.5, 824.0, 761.7, 604.0 cm^–1^; APCI-MS *m/z*: 342.02[M+H]^+^. Anal. calcd for C_19_H_19_NO_5_: C 66.85, H 5.61, N 4.10; found C 66.90, H 5.60, N 4.11.

***3-allyloxy-2-((dimethylamino)methyl)-1-hydroxy-9H-xanthen-9-one(3a)***

Compoun-d **3** (0.268 g, 1 mmol) and a solution of dimethylamine (2 mL, 33%, 8.7 mmol) were reacted as described above. The crude product was purified by column chromatography eluting with chloroform: methanol: ammonia = 40: 1: 0.5 to yield compound **3a** as a yellow solid. Yield: 21.5%; m.p. 114~118 ˚C; ^1^H NMR (CDCl_3_, 500 MHz) *δ*: 8.27 (d, *J*=7.8 Hz, 1H, H-8), 7.71 (t, *J*=8.3 Hz, 1H, H-6), 7.42 (d, *J*=8.4 Hz, 1H, H-5), 7.38 (t, *J*=7.6 Hz, 1H, H-7), 6.45 (s, 1H, H-4), 6.06~6.12 (m, 1H, C**H**=), 5.49 (d, *J*=17.3 Hz, 1H, =C**H**_2_), 5.35 (d, *J*=10.6 Hz, 1H, =C**H**_2_), 4.48 (d, *J*=4.9 Hz, 2H, OC**H**_2_), 3.60 (s, 2H, C**H**_2_N), 2.33 (s, 6H, 2C**H**_3_); ^13^C NMR (CDCl_3_, 125 MHz) *δ*: 179.50 (C-9), 163.07 (C-3), 160.38 (C-1), 155.90 (C-4a), 154.68 (C-4b), 133.64 (C-6), 131.01 (CH_2_**C**H), 124.75 (C-8), 122.80 (C-7), 119.60 (C-8a), 116.79 (**C**H_2_=CH), 116.29 (C-5), 107.91 (C-8b), 102.62 (C-2), 89.55 (C-4), 68.19 (O**C**H_2_), 48.60 (**C**H_3_), 44.36 (**C**H_2_N); IR (KBr) *ν*: 3446, 3079, 2969, 2941, 2856, 2814, 2772, 2754, 1644, 1607, 1569, 1480, 1468, 1387, 1371, 1354, 1319, 1277, 1258, 1228, 1204, 1182, 1159, 1133, 1094, 1036, 1020, 923, 877, 847, 815, 760, 669, 596 cm^–1^; ESI-MS *m/z*: 326.05[M+H]^+^. Anal. calcd for C_19_H_19_NO_4_: C 70.14, H 5.89, N 4.31; found C 70.00, H 5.5.88, N 4.30.

***3-allyloxy-2-((diethylamino)methyl)-1-hydroxy-9H-xanthen-9-one (3b)***

Compound **3** (0.268 g, 1 mmol) and diethylamine (1 mL, 9.70mmol) were reacted as described above. The crude product was purified by column chromatography eluting with chloroform: methanol: ammonia = 40: 1: 0.5 to yield compound **3b** as a yellow solid. Yield: 34%; m.p. 113~115 ˚C; ^1^H NMR (CDCl_3_, 500 MHz) *δ*: 8.28 (d, *J*=8.0 Hz, 1H, H-8), 7.71 (t, *J*=7.2 Hz, 1H, H-6), 7.42 (d, *J*=8.4 Hz, 1H, H-5), 7.38 (t, *J*=7.4 Hz, 1H, H-7), 6.44 (s, 1H, H-4), 6.07~6.15 (m, 1H, C**H**=), 5.49 (d, *J*=17.2 Hz, 1H, =C**H**_2_), 5.36 (d, *J*=10.5 Hz, 1H, =C**H**_2_), 4.68 (d, *J*=5.2 Hz, 2H, OC**H**_2_), 3.66 (s, 2H, C**H**_2_N), 2.67 (q, 4H, 2C**H**_2_), 1.13 (t, *J*=7.1 Hz, 6H, 2C**H**_3_); ^13^C NMR (CDCl_3_, 125 MHz) *δ*: 180.32 (C-9), 164.18 (C-3), 161.78 (C-1), 157.20 (C-4a), 155.84 (C-4b), 134.71 (C-6), 132.31 (CH_2_**C**H), 126.04 (C-8), 123.94 (C-7), 121.02 (C-8a), 118.39 (**C**H_2_=CH), 117.44 (C-5), 109.14 (C-8b), 104.07 (C-2), 90.79 (C-4), 69.62 (O**C**H_2_), 46.86 (**C**H_2_CH_3_), 43.91 (**C**H_2_N), 11.16 (**C**H_3_); IR (KBr) *ν*: 3789, 3424, 2963, 2346, 1644, 1607, 1570, 1468, 1317, 1259, 1230, 1201, 1137, 1094, 816 cm^–1^; ESI-MS *m/z*: 354.07[M+H]^+^. Anal. calcd for C_21_H_23_NO_4_: C 71.37, H 6.56, N 3.96; found C 71.28, H 6.56, N 3.95.

***3-allyloxy-1-hydroxy-2-(pyrrolidin-1-ylmethyl)- 9H-xanthen-9-one (3c)***

Compound **3** (0.268 g, 1 mmol) and pyrrolidine (1 mL, 12.1 mmol) were reacted as described above. The crude product was purified by column chromatography eluting with chloroform: methanol: ammonia = 40: 1: 0.5 to yield compound **3c** as a yellow solid. Yield: 42.7%; m.p. 144~148 ˚C; ^1^H NMR (CDCl_3_, 500 MHz) *δ*: 8.26 (dd, *J*=1.4, 8.0 Hz, 1H, H-8), 7.71 (td, *J*=1.6, 8.6 Hz, 1H, H-6), 7.42 (d, *J*=8.3 Hz, 1H, H-5), 7.38 (t, *J*=7.8 Hz, 1H, H-7), 6.44 (s, 1H, H-4), 6.06~6.12 (m, 1H, C**H**=), 5.50 (dd, *J*=1.2, 17.2 Hz, 1H, =C**H**_2_), 5.35 (dd, *J*=1.0, 10.5 Hz, 1H, =C**H**_2_), 4.68 (d, *J*=5.0 Hz, 2H, OC**H**_2_), 3.85 (s, 2H, C**H**_2_N), 2.67 (s, 4H, H-1' and 4'), 1.78 (s, 4H, H-2' and 3'); ^13^C NMR (CDCl_3_, 125 MHz) *δ*: 180.59 (C-9), 164.10 (C-3), 161.43 (C-1), 156.99 (C-4a), 155.84 (C-4b), 134.78 (C-6), 132.22 (CH_2_**C**H), 125.92 (C-8), 123.95 (C-7), 120.79 (C-8a), 117.98 (**C**H_2_=CH), 117.46 (C-5), 109.11 (C-8b), 103.85 (C-2), 90.66 (C-4), 69.32 (O**C**H_2_), 53.98 (C-1' and 4'), 45.60 (**C**H_2_N), 23.50 (C-2' and 3'); IR (KBr) *ν*: 3428, 2944, 2805, 1650, 1609, 1485, 1469, 1377, 1319, 1265, 1229, 1209, 1145, 1124, 930, 877, 836, 799, 750, 616 cm^–1^; ESI-MS *m/z*: 352.01[M+H]^+^. Anal. calcd for C_21_H_21_NO_4_: C 71.78, H 6.02, N 3.99; found C 71.85, H 6.03, N 4.00.

***3-allyloxy-1-hydroxy-2-(piperidin-1-ylmethyl)-9H-xanthen-9-one(3d)***

Compound **3** (0.268 g, 1 mmol) and piperidine (1 mL, 10.1 mmol) were reacted as described above. The crude product was purified by column chromatography eluting with chloroform: methanol: ammonia = 40: 1: 0.5 to yield compound **3d** as a yellow solid. Yield: 27.4%; m.p. 158~164 ˚C; ^1^H NMR (CDCl_3_, 500 MHz) *δ*: 8.28 (d, *J*=7.9 Hz, 1H, H-8), 7.72 (t, *J*=7.3 Hz, 1H, H-6), 7.44 (d, *J*=8.4 Hz, 1H, H-5), 7.39 (t, *J*=7.5 Hz, 1H, H-7), 6.46 (s, 1H, H-4), 6.07~6.13 (m, 1H, C**H**=), 5.52 (d, *J*=17.3 Hz, 1H, =C**H**_2_), 5.37 (d, *J*=10.5 Hz, 1H, =C**H**_2_), 4.68 (d, *J*=1.0 Hz, 2H, OC**H**_2_), 3.73 (s, 2H, C**H**_2_N), 2.57 (brs, 4H, H-1' and 5'), 1.61 (brs, 4H, H-2' and 4'), 1.42 (brs, 2H, H-3'); ^13^C NMR (CDCl_3_, 125 MHz) *δ*: 180.45 (C-9), 164.31 (C-3), 161.71 (C-1), 157.07 (C-4a), 155.82 (C-4b), 134.74 (C-6), 132.23 (CH_2_**C**H), 125.97 (C-8), 123.94 (C-7), 120.90 (C-8a), 117.96 (**C**H_2_=CH), 117.44 (C-5), 108.37 (C-8b), 103.92 (C-2), 90.69 (C-4), 69.31 (O**C**H_2_), 54.32 (C-1' and 5'), 49.62 (**C**H_2_N), 25.97 (C-2' and 4'), 24.25 (C-3'); IR (KBr) *ν*: 3426, 3071, 2936, 2853, 2796, 2763, 2346, 1646, 1607, 1483, 1469, 1403, 1319, 1295, 1275, 1251, 1227, 1208, 1149, 1134, 1109, 1093, 1036, 1012, 986, 926, 845, 756 cm^–1^; ESI-MS *m/z*: 366.07[M+H]^+^. Anal. calcd for C_16_H_15_NO_4_: C 72.31, H 6.34, N 3.83; found C 72.15, H 6.35, N 3.83.

***3-allyloxy-1-hydroxy-2-(morpholinomethyl)-9H-xanthen-9-one(3e)***

Compound **3** (0.268 g, 1 mmol) and morpholine (1 mL, 17.2 mmol) were reacted as described above. The crude product was purified by column chromatography eluting with chloroform: methanol: ammonia = 40: 1: 0.5 to yield compound **3e** as a yellow solid. Yield: 13.6%; m.p. 188~193 ˚C; ^1^H NMR (CDCl_3_, 500 MHz) *δ*: 8.28 (dd, *J*=1.5, 7.9 Hz, 1H, H-8), 7.74 (td, *J*=1.5, 8.6 Hz, 1H, H-6), 7.45 (d, *J*=8.4 Hz, 1H, H-5), 7.40 (t, *J*=7.7 Hz, 1H, H-7), 6.46 (s, 1H, H-4), 6.06~6.13 (m, 1H, C**H**=), 5.52 (dd, *J*=1.2, 17.2 Hz, 1H, =C**H**_2_), 5.38 (dd, *J*=1.3, 10.8 Hz, 1H, =C**H**_2_), 4.69 (d, *J*=3.8 Hz, 2H, OC**H**_2_), 3.71~3.75 (m, 6H, C**H**_2_N, H-2' and 4'), 2.61 (t, *J*=4.5 Hz, 4H, H-1' and 5'); ^13^C NMR (CDCl_3_, 125 MHz) *δ*: 180.72 (C-9), 164.39 (C-3), 161.67 (C-1), 157.15 (C-4a), 155.87 (C-4b), 134.92 (C-6), 132.10 (CH_2_**C**H), 125.93 (C-8), 124.08 (C-7), 120.75 (C-8a), 118.11 (**C**H_2_=CH), 117.51 (C-5), 107.88 (C-8b), 103.77 (C-2), 90.76 (C-4), 69.35 (O**C**H_2_), 67.14 (C-2' and 4'); 53.47 (C-1' and 5'), 49.06 (**C**H_2_N); IR (KBr) *ν*: 3428, 2854, 1655, 1608, 1570, 1466, 1318, 1287, 1232, 1141, 1114, 863 cm^–1^; APCI-MS *m/z*: 368.03[M+H]^+^. Anal. calcd for C_21_H_21_NO_5_: C 68.65, H 5.76, N 3.81; found C 66.68, H 5.75, N 3.80.

***2-((dimethylamino)methyl)-1-hydroxy-3-(3-methylbut-2-enyloxy)-9H-xanthen-9-one (4a)***

Compound **4** (0.296 g, 1 mmol) and a solution of dimethylamine (2 mL, 33%, 8.7 mmol) were reacted as described above. The crude product was purified by column chromatography eluting with chloroform: methanol: ammonia = 40: 1: 0.5 to yield compound **4a** as a yellow solid. Yield: 32.0%; m.p. 122~124 ˚C; ^1^H NMR (CDCl_3_, 500 MHz) *δ*: 8.29 (dd, *J*=1.4, 7.9 Hz, 1H, H-8), 7.72 (td, *J*=1.5, 8.5 Hz, 1H, H-6), 7.44 (d, *J*=8.4 Hz, 1H, H-5), 7.39 (t, *J*=7.7 Hz, 1H, H-7), 6.47 (s, 1H, H-4), 5.53 (t, *J*=6.5 Hz, 1H, C**H**=), 4.68 (d, *J*=6.8 Hz, 2H, OC**H**_2_), 3.59 (s, 2H, C**H**_2_N), 2.34 (s, 6H, 2NC**H**_3_), 1.84 (s, 3H, =C-C**H**_3_), 1.79 (s, 3H, =C-C**H**_3_); ^13^C NMR (CDCl_3_, 125 MHz) *δ*: 180.60 (C-9), 164.71 (C-3), 161.49 (C-1), 157.09 (C-4a), 155.85 (C-4b), 138.31 (C-6), 134.76 (CH_2_**C**H), 125.91 (C-8), 123.93 (C-7), 120.80 (C-8a), 118.95 (**C**H_2_=CH), 117.46 (C-5), 109.00 (C-8b), 103.64 (C-2), 90.67 (C-4), 65.85 (O**C**H_2_), 49.71 (N**C**H_3_), 45.52 (**C**H_2_N), 25.82 (**C**H_3_), 18.41 (**C**H_3_); IR (KBr) *ν*: 3429, 3089, 2962, 2936, 2855, 2815, 2770, 2756, 1644, 1607, 1568, 1480, 1461, 1439, 1397, 1383, 1369, 1341, 1317, 1275, 1259, 1229, 1206, 1182, 1159, 1133, 1093, 1036, 1019, 1002, 975, 877, 846, 819, 776, 761, 617 cm^–1^; APCI-MS *m/z*: 354.06[M+H]^+^. Anal. calcd for C_21_H_23_NO_4_: C 71.37, H 6.56, N 3.96; found C 71.40, H 6.57, N 3.97.

***2-((diethylamino)methyl)-1-hydroxy-3-(3-methylbut-2-enyloxy)-9H-xanthen-9-one (4b)***

Compound **4** (0.296 g, 1 mmol) and diethylamine (1 mL, 9.70mmol) were synthesized reacted as described above. The crude product was purified by column chromatography eluting with chloroform: methanol: ammonia = 40: 1: 0.5 to yield compound **4b** as a yellow solid. Yield: 35.5%; m.p. 105~107 ˚C; ^1^H NMR (CDCl_3_, 500 MHz) *δ*: 8.29 (dd, *J*=1.4, 7.9 Hz, 1H, H-8), 7.71 (td, *J*=1.7, 8.6 Hz, 1H, H-6), 7.43 (d, *J*=8.3 Hz, 1H, H-5), 7.38 (td, *J*=0.7, 7.9 Hz, 1H, H-7), 6.46 (s, 1H, H-4), 5.53 (t, *J*=6.6 Hz, 1H, C**H**=), 4.66 (d, *J*=6.6 Hz, 2H, OC**H**_2_), 3.72 (s, 2H, C**H**_2_N), 2.66 (q, 4H, 2C**H**_2_CH_3_), 1.84 (s, 3H, =C-C**H**_3_), 1.79 (s, 3H, =C-C**H**_3_), 1.13 (t, 6H, *J*=7.1 Hz, 2C**H**_3_) ; ^13^C NMR (CDCl_3_, 125 MHz) *δ*: 180.16 (C-9), 164.60 (C-3), 161.60 (C-1), 157.05 (C-4a), 155.76 (C-4b), 138.41 (C-6), 134.54 (CH_2_**C**H=), 125.96 (C-8), 123.70 (C-7), 121.00 (C-8a), 118.92 (**C**H_2_=CH), 117.36 (C-5), 109.54 (C-8b), 103.87 (C-2), 90.60 (C-4), 65.70 (O**C**H_2_), 46.81 (**C**H_2_CH_3_), 43.67 (**C**H_2_N), 25.75 (=CH_2_**C**H_3_), 18.31 (=CH_2_**C**H_3_), 11.28 (**C**H_3_); IR (KBr) *ν*: 3425, 2963, 2344, 1653, 1608, 1569, 1483, 1467, 1398, 1384, 1313, 1268, 1233, 1192, 1165, 1146, 1120, 1091, 1048, 982, 814, 782, 757, 612 cm^–1^; APCI-MS *m/z*: 382.09[M+H]^+^. Anal. calcd for C_23_H_27_NO_4_: C 72.42, H 7.13, N 3.67; found C 72.36, H 7.13, N 3.68.

***1-hydroxy-3-(3-methylbut-2-enyloxy)-2-(pyrrolidin-1-ylmethyl)-9H-xanthen-9-one (4c)***

Compound **4** (0.296 g, 1 mmol) and pyrrolidine (1 mL, 12.1 mmol) were reacted as described above. The crude product was purified by column chromatography eluting with chloroform: methanol: ammonia = 40: 1: 0.5 to yield compound **4c** as a yellow solid. Yield: 42.7%, m.p. 151~153 ˚C; ^1^H NMR (CDCl_3_, 500 MHz) *δ*: 8.29 (dd, *J*=1.5, 8.0 Hz, 1H, H-8), 7.72 (td, *J*=1.6, 8.6 Hz, 1H, H-6), 7.44 (d, *J*=8.4 Hz, 1H, H-5), 7.39 (t, *J*=7.3 Hz, 1H, H-7), 6.47 (s, 1H, H-4), 5.53 (t, *J*=6.5 Hz, 1H, C**H**=), 4.67 (d, *J*=6.5 Hz, 2H, OC**H**_2_), 3.82 (s, 2H, C**H**_2_N), 2.66 (s, 4H, H-1' and 4'), 1.84 (s, 3H, =C-C**H**_3_), 1.76~1.80 (m, 7H, =C-C**H**_3_, H-2' and 3'); ^13^C NMR (CDCl_3_, 125 MHz) *δ*: 180.50 (C-9), 164.57 (C-3), 161.37 (C-1), 156.99 (C-4a), 155.83 (C-4b), 138.33 (C-6), 134.68 (CH_2_**C**H), 125.90 (C-8), 123.87 (C-7), 120.82 (C-8a), 118.92 (**C**H_2_=CH), 117.42 (C-5), 109.04 (C-8b), 103.68 (C-2), 90.58 (C-4), 65.74 (O**C**H_2_), 53.88 (C-1' and 4'), 45.47 (**C**H_2_N), 25.77 (=CH_2_**C**H_3_), 23.49 (C-2' and 3'), 18.35 (=CH_2_**C**H_3_); IR (KBr) *ν*: 3791, 3431, 3069, 2967, 2947, 2873, 2814, 2783, 2238, 1643, 1610, 1496, 1485, 1463, 1443, 1404, 1375, 1316, 1262, 1228, 1213, 1139, 1123, 1088, 1046, 1032, 1009, 975, 952, 928, 876, 838, 800, 787, 750, 611, 557 cm^–1^; APCI-MS *m/z*: 380.09[M+H]^+^. Anal. calcd for C_23_H_25_NO_4_: C 72.80, H 6.64, N 3.69; found C 72.77, H 6.64, N 3.70.

***1-hydroxy-3-(3-methylbut-2-enyloxy)-2-(piperidin-1-ylmethyl)-9H-xanthen-9-one (4d)***

Compound **4** (0.296 g, 1 mmol) and piperidine (1 mL, 10.1 mmol) were reacted as described above. The crude product was purified by column chromatography eluting with chloroform: methanol: ammonia = 40: 1: 0.5 to yield compound **4d** as a yellow solid. Yield: 45.5%, m.p. 166~168 ˚C; ^1^H NMR (CDCl_3_, 500 MHz) *δ*: 8.29 (dd, *J*=1.5, 8.0 Hz, 1H, H-8), 7.72 (td, *J*=1.6, 8.6 Hz, 1H, H-6), 7.44 (d, *J*=8.4 Hz, 1H, H-5), 7.39 (t, *J*=7.6 Hz, 1H, H-7), 6.46 (s, 1H, H-4), 5.52 (t, *J*=6.5 Hz, 1H, C**H**=), 4.65 (d, *J*=6.5 Hz, 2H, OC**H**_2_), 3.71 (s, 2H, C**H**_2_N), 2.55 (brs, 4H, H-1' and 5'), 1.85 (s, 3H, =C-C**H**_3_), 1.80 (s, 3H, =C-C**H**_3_), 1.57~1.62 (m, 4H, H-2' and 4'), 1.41 (brs, 2H, H-3'); ^13^C NMR (CDCl_3_, 125 MHz) *δ*: 180.34 (C-9), 164.76 (C-3), 161.63 (C-1), 157.06 (C-4a), 155.80 (C-4b), 138.51 (C-6), 134.63 (CH_2_**C**H), 125.94 (C-8), 123.85 (C-7), 120.92 (C-8a), 118.88 (**C**H_2_=CH), 117.39 (C-5), 108.31 (C-8b), 103.77 (C-2), 90.57 (C-4), 65.68 (O**C**H_2_), 54.20 (C-1' and 5'), 49.52 (**C**H_2_N), 26.02 (C-2' and 4'), 25.77 (=CH_2_**C**H_3_), 24.25 (C-3'), 18.34 (=CH_2_**C**H_3_); IR (KBr) *ν*: 3424, 3071, 2965, 2937, 2927, 2852, 2818, 2758, 1646, 1619, 1607, 1484, 1468, 1401, 1387, 1317, 1295, 1271, 1249, 1226, 1209, 1149, 1132, 1110, 1092, 1035, 1010, 987, 970, 875, 845, 809, 756, 613 cm^–1^; APCI-MS *m/z*: 394.08[M+H]^+^. Anal. calcd for C_24_H_27_NO_4_: C 73.26, H 6.92, N 3.56; found C 73.35, H 6.93, N 3.57.

***1-hydroxy-3-(3-methylbut-2-enyloxy)-2-morpholinomethyl)-9H-xanthen-9-one (4e)***

Compound **4** (0.296 g, 1 mmol) and morpholine (1 mL, 17.2 mmol) were reacted as described above. The crude product was purified by column chromatography eluting with chloroform: methanol: ammonia = 40: 1: 0.5 to yield compound **4e** as a yellow solid. Yield: 16.6%, m.p. 135~138 ˚C; ^1^H NMR (CDCl_3_, 500 MHz) *δ*: 8.29 (dd, *J*=1.5, 8.0 Hz, 1H, H-8), 7.74 (td, *J*=1.7, 8.6 Hz, 1H, H-6), 7.45 (d, *J*=8.3 Hz, 1H, H-5), 7.40 (td, *J*=0.7, 7.9 Hz, 1H, H-7), 6.48 (s, 1H, H-4), 5.52 (t, *J*=6.6 Hz, 1H, C**H**=), 4.66 (d, *J*=6.5 Hz, 2H, OC**H**_2_), 3.71~3.74 (m, 6H, C**H**_2_N, H-2' and 4'), 2.60 (t, *J*=4.5 Hz, 4H, H-1' and 5'), 1.85 (s, 3H, =C-C**H**_3_), 1.80 (s, 3H, =C-C**H**_3_); ^13^C NMR (CDCl_3_, 125 MHz) *δ*: 180.67 (C-9), 164.87 (C-3), 161.62 (C-1), 157.18 (C-4a), 155.87 (C-4b), 138.78 (C-6), 134.85 (CH_2_**C**H), 125.93 (C-8), 124.02 (C-7), 120.77 (C-8a), 118.71 (**C**H_2_=CH), 117.49 (C-5), 107.74 (C-8b), 103.63 (C-2), 90.66 (C-4), 67.16 (O**C**H_2_), 65.75 (C-2' and 4'); 53.39 (C-1' and 5'), 48.96 (**C**H_2_N), 25.82 (=CH_2_**C**H_3_), 18.39 (=CH_2_**C**H_3_); IR (KBr) *ν*: 3440, 2925, 2856, 1651, 1608, 1482, 1466, 1404, 1385, 1314, 1287, 1229, 1205, 1162, 1138, 1115, 1092, 1033, 1001, 863, 828, 788, 757, 610, 490 cm^–1^; APCI-MS *m/z*: 396.05[M+H]^+^. Anal. calcd for C_23_H_25_NO_5_: C 69.86, H 6.37, N 3.54; found C 69.90, H 6.38, N 3.55 .
